# TGF-β2 mediated secretion of sCTLA-4 from regulatory T cells

**DOI:** 10.1186/1476-9255-12-S1-P6

**Published:** 2015-04-16

**Authors:** R Khanolkar, S Rajpara, F Muller, I Depasquale, L Lawson, RN Barker, M Nicolson, AD Ormerod, FJ Ward

**Affiliations:** 1 Division of Applied Medicine, Institute of Medical Sciences, Foresterhill, Aberdeen AB25 2ZD UK; 2 Department of Dermatology, Aberdeen Royal Infirmary, Aberdeen AB25 2ZR UK; 3 Department of Plastic surgery, Aberdeen Royal Infirmary, Foresterhill, Aberdeen AB25 2ZN UK; 4 Department of Oncology, Aberdeen Royal Infirmary, Foresterhill, Aberdeen AB25 2ZN UK

## 

Cytotoxic T lymphocyte antigen-4 (CTLA-4), a membrane bound inhibitory receptor whose expression is induced on activated T cells, has been established as an important regulator of T cell responses and serves to maintain peripheral tolerance. Additionally, recent characterization of a novel mechanism of extrinsic suppression mediated by the soluble isoform of CTLA-4 (sCTLA-4) has served to further establish the importance of CTLA-4 in maintaining homeostasis of the immune system. Previously, we have shown that selective blockade of sCTLA-4 in patients with metastatic melanoma enhances immune responses including increased antigen-specific proliferation of both CD4+ and CD8+ T cells, compared with an isotype antibody control or pan CTLA-4 blockade. Selective blockade also enhanced T cell effector cytokine responses, notably IFN-γ and IL-17. In this study, we demonstrate a protocol for generating a regulatory T cell population, which is characterised by the production of high levels of sCTLA-4. Treatment of naïve human T cells with TGF-β2 together with an anti-CD3 mAb/IL-2 T cell stimulus increased their capacity to produce high levels of sCTLA-4, while decreasing production of effector cytokines - IFN-γ, IL-17 and IL-10. Furthermore, analysis of these sCTLA-4 producing T cells has shown that they express the T regulatory cell transcription factor – FoxP3. Taken together, our data show that TGF-β2 could serve as an attractive therapeutic tool to alleviate symptoms of autoimmunity through the induction of regulatory T cell populations that secrete immunosuppressive sCTLA-4; while on the other hand, neutralizing the effects of TGF-β2 could also prove beneficial to patients with cancer.

